# Codelivery of Chemotherapeutics via Crosslinked Multilamellar Liposomal Vesicles to Overcome Multidrug Resistance in Tumor

**DOI:** 10.1371/journal.pone.0110611

**Published:** 2014-10-17

**Authors:** Yarong Liu, Jinxu Fang, Kye-Il Joo, Michael K. Wong, Pin Wang

**Affiliations:** 1 Mork Family Department of Chemical Engineering and Materials Science, University of Southern California, Los Angeles, California, United States of America; 2 Division of Medical Oncology, Norris Comprehensive Cancer Center, Keck School of Medicine, University of Southern California, Los Angeles, California, United States of America; 3 Department of Biomedical Engineering, University of Southern California, Los Angeles, California, United States of America; 4 Department of Pharmacology and Pharmaceutical Sciences, University of Southern California, Los Angeles, California, United States of America; Brandeis University, United States of America

## Abstract

Multidrug resistance (MDR) is a significant challenge to effective cancer chemotherapy treatment. However, the development of a drug delivery system that allows for the sustained release of combined drugs with improved vesicle stability could overcome MDR in cancer cells. To achieve this, we have demonstrated codelivery of doxorubicin (Dox) and paclitaxel (PTX) *via* a crosslinked multilamellar vesicle (cMLV). This combinatorial delivery system achieves enhanced drug accumulation and retention, in turn resulting in improved cytotoxicity against tumor cells, including drug-resistant cells. Moreover, this delivery approach significantly overcomes MDR by reducing the expression of P-glycoprotein (P-gp) in cancer cells, thus improving antitumor activity *in vivo*. Thus, by enhancing drug delivery to tumors and lowering the apoptotic threshold of individual drugs, this combinatorial delivery system represents a potentially promising multimodal therapeutic strategy to overcome MDR in cancer therapy.

## Introduction

The development of multidrug resistance (MDR) against a variety of conventional and novel chemotherapeutic agents has been a major impediment to the success of cancer therapy [1], [2]. One of the most important mechanisms involved in MDR is the overexpression of P-glycoprotein (P-gp) in the plasma membrane of various cancer cells. P-gp, an active drug efflux transporter, is capable of effluxing a broad range of anticancer agents, such as taxanes and anthracyclines [3]. For example, the efficacy of doxorubicin (Dox) and paclitaxel (PTX), two of the most widely used agents for the treatment of various cancers, is often compromised by P-gp-mediated MDR [4], [5]. Therefore, a strategy to inhibit P-gp expression has been developed to overcome MDR. For instance, a large number of P-gp inhibitors and siRNAs targeting the gene encoding P-gp have been delivered in combination with anticancer agents to downregulate P-gp expression, thereby enabling drugs to reach sufficient concentrations to induce cytotoxicity [6], [7]. However, P-gp inhibitors, either functional inhibitors or siRNA, have yielded disappointing clinical trials resulting from their high systemic toxicities and enhanced side effects of chemotherapy in normal cells [8], [9].

Combination therapy with multiple chemotherapeutics provides an alternative strategy to suppress MDR. Different drugs may attack cancer cells at varying stages of their growth cycles, thus decreasing the concentration threshold for individual drugs that is otherwise required for cytotoxicity [10]. It has been reported that various drug combinations have successfully induced synergistic antitumor activities and prevented disease recurrence [11], [12]. For example, a Dox and PTX cocktail is now considered a standard anthracycline-taxane combination treatment for various tumors by their ability to overcome drug resistance [13], [14], [15]. However, a major challenge of combination therapy is coordinating the pharmacokinetics and cellular uptake of combined therapeutics. This obstacle has limited the clinical success of combination therapy [16], [17].

To overcome this challenge, novel strategies that allow loading of multiple therapeutics into a single drug-delivery vehicle for concurrent delivery at the site of action have been extensively explored [18], [19]. Several drug delivery systems have been able to intercalate multiple drugs for site-specific delivery to tumors and, hence, improve antitumor activities, potentially overcoming drug resistance, while, at the same time, reducing the dosage of individual drugs [20], [21], [22]. Indeed, nanoparticle delivery systems are known to deliver therapeutics efficiently to the tumor sites through the enhanced permeability and retention (EPR) effect, thereby enhancing the concentration of therapeutics in tumors [23], [24]. Moreover, these nanoparticles can enter cancer cells through endocytosis in a manner independent of the P-gp pathway, thereby enhancing cellular accumulation of therapeutics [25], [26], [27]. Thus, a nanoparticle delivery system capable of mediating high efficiency of cellular entry and subsequent triggering of intracellular release of multiple anticancer drugs to overcome MDR is highly desirable.

Liposomes are one of the most popular nanoparticle delivery systems for combinatorial delivery of multiple drugs based on their ability to efficiently load both hydrophilic and hydrophobic drugs [24], [28]. We previously developed a robust crosslinked multilamellar liposomal vesicle (cMLV), with enhanced vesicle stability, to efficiently codeliver hydrophilic (Dox) and hydrophobic (PTX) drugs and induce ratio-dependent synergistic antitumor activity, both *in vitro* and *in vivo* [29], [30], [31]. Moreover, it was shown that cMLV particles are mainly internalized by cells through caveolin-dependent endocytosis and are then trafficked through the endosome-lysosome network for release of drugs [30]. In this study, we have examined the potential of cMLV as a combinatorial delivery system aimed at overcoming P-gp-mediated drug resistance, both *in vitro* and *in vivo*. Indeed, we have demonstrated that the combination of Dox and PTX, when administered at 1∶1 weight ratio in cMLV formulations, shows significant enhancement of cytotoxicity and antitumor activities. Combining these drugs through the use of cMLV formulations contributes to these antitumor activities by enhancing systemic delivery efficiency and lowering tumor apoptotic threshold.

## Materials and Methods

### Mice

Female BALB/c mice (6–10 weeks old) were purchased from Charles River Breeding Laboratories (Wilmington, MA). All mice were held under specific pathogen-reduced conditions in the Animal Facility of the University of Southern California (USA). All experiments were performed in accordance with the guidelines set by the National Institutes of Health and the University of Southern California on the Care and Use of Animals. This study was approved by the Committee on the Ethics of Animal Experiments of the University of Southern California.

### Cell culture

B16 tumor cells (B16–F10, ATCC number: CRL-6475) and 4T1 tumor cells (ATCC number: CRL-2539) were maintained in a 5% CO_2_ environment with Dulbecco’s modified Eagle’s medium (Mediatech, Inc., Manassas, VA) supplemented with 10% FBS (Sigma-Aldrich, St. Louis, MO) and 2 mM of L-glutamine (Hyclone Laboratories, Inc., Omaha, NE). B16-R and 4T1-R cells were produced by continuously treating B16 and 4T1 cells with 5 µg/ml PTX for 4 days. The cells were then recovered by replacing medium with fresh medium without drugs for 7 days. The remaining cells formed drug resistance for PTX. JC cells (ATCC number: CRL-2116) were used as a model drug-resistant tumor cell line because it has been shown that JC cells overexpress P-gp and exhibit a drug-resistant phenotype, both *in vitro* and *in vivo* [32].

### Synthesis of cMLVs

Liposomes were prepared based on the conventional dehydration-rehydration method. All lipids were obtained from the NOF Corporation (Japan). 1.5 µmol of lipids 1,2-dioleoyl-sn-glycero-3-phosphocholine (DOPC), 1,2-dioleoyl-sn-glycero-3-phospho-(1′-rac-glycerol) (DOPG), and maleimide-headgrouplipid1,2-dioleoyl-sn-glycero-3-phosphoeth-anolamine-N-[4-(p-maleimidophenyl) butyramide (MPB-PE) were combined in chloroform at a molar lipid ratio of DOPC:DOPG:MPB = 4∶1∶5, and the organic solvent in the lipid mixture was evaporated under argon gas. The lipid mixture was further dried under vacuum overnight to form dried thin lipid films. To prepare cMLV(PTX) and cMLV(Dox+PTX) at a molar ratio of 0.2∶1 (drugs:lipids), paclitaxel in organic solvent was mixed with the lipid mixture to form dried thin lipid films. The resultant dried film was hydrated in 10 mM Bis-Tris propane at pH 7.0 with (cMLV(Dox) or cMLV(Dox+PTX)) or without doxorubicin (cMLV(PTX)) at a molar ratio of 0.2∶1 (drugs:lipids) with vigorous vortexing every 10 min for 1 h, followed by applying 4 cycles of 15-s sonication (Misonix Microson XL2000, Farmingdale, NY) on ice in 1-min intervals of each cycle. To induce divalent-triggered vesicle fusion, MgCl_2_ was added at a final concentration of 10 mM. The resulting multilamellar vesicles were further crosslinked by addition of Dithiothreitol (DTT, Sigma-Aldrich) at a final concentration of 1.5 mM for 1 h at 37°C. The resulting vesicles were collected by centrifugation at 14,000 g for 4 min and then washed twice with PBS. For pegylation of cMLVs, the particles were incubated with 1 µmol of 2 kDa PEG-SH (Laysan Bio Inc., Arab, AL) for 1 h at 37°C. The particles were then centrifuged and washed twice with PBS. The final products were stored in PBS at 4°C. The mean diameter of all cMLVs is around 220 nm determined by dynamic light scattering (DLS), and around 160 nm estimated by cryo-electron microscopy. The loading efficiency, and stability of cMLVs were similar to that demonstrated previously [30], [31].

### *In vitro* cytotoxicity and data analysis

B16–F10, 4T1, B16–R, 4T1-R, and JC cells were plated at a density of 5×10^3^ cells per well in D10 media in 96-well plates and grown for 6 h. The cells were then exposed to a series of concentrations of cMLV (single drug) or cMLV (drug combinations) for 48 h. The cell viability was assessed using the Cell Proliferation Kit II (XTT assay) from Roche Applied Science according to the manufacturer’s instructions. Slope m and IC_50_ were obtained from median effect model, and IIP_Cmax_ was calculated via the following equation: IIP_Cmax_ = log (1+(Cmax/IC_50_)^m^). Cmax is the maximum plasma drug concentrations for the commonly recommended dose for each drug.

### Cellular uptake of doxorubicin and paclitaxel in cells

4T1 cells were seeded in 24-well plates at a density of 2×10^5^ cells per well and grown overnight. The cells were then exposed to empty cMLVs (control), cMLV(Dox), cMLV(PTX), cMLV(Dox+PTX), and Dox+PTX. The final concentrations of Dox and PTX were 1 µg/ml for each group. JC cells were seeded at a density of 10^5^ cells per well in D10 media in 96-well plates. The cells were exposed to empty cMLVs, cMLV(Dox), cMLV(PTX), cMLV(Dox+PTX), and Dox+PTX. The final concentrations of Dox and PTX were 5 µg/ml for each group. At 48 h after treatment, the cells were washed twice with PBS and lysed with PBS containing 1% Triton X-100. Doxorubicin and paclitaxel in cell lysates were extracted by 1∶1 (v/v) Chloroform/isopropyl alcohol or ethyl acetate, respectively. Paclitaxel concentrations in cell lysates were measured by HPLC C18 column and detected at 227 nm (flow rate 1****ml/min), and doxorubicin was detected by fluorescence with 480/550 nm excitation/emission. The concentrations of Dox and PTX were normalized for protein content as measured with BCA assay (Pierce).

### *In vivo* antitumor activity study

BALB/c female mice (6–10 weeks old) were inoculated subcutaneously with 0.2×10^6^ 4T1 breast tumor cells. The tumors were allowed to grow for 8 days to a volume of ∼50 mm^3^ before treatment. After 8 days, the mice were injected intravenously through the tail vein with cMLV(2****mg/kg Dox), cMLV(2****mg/kg PTX), cMLV(2****mg/kg Dox)+cMLV(2****mg/kg PTX), or cMLV(2****mg/kg Dox + 2****mg/kg PTX) every three days (six mice per group). Tumor growth and body weight were monitored for 40 days or to the end of the experiment. The length and width of the tumor masses were measured with a fine caliper every three days after injection. Tumor volume was expressed as 1/2 × (length × width^2^). Survival end point was set when the tumor volume reached 1000 mm^3^. The survival rates are presented as Kaplan-Meier curves. The survival curves of individual groups were compared by a log-rank test.

### Immunohistochemistry of tumors and confocal imaging

BALB/c female mice (6–10 weeks old) were inoculated subcutaneously with 0.2 × 10^6^ 4T1 or JC tumor cells. The tumors were allowed to grow for 20 days to a volume of ∼500 mm^3^ before treatment. On day 20, the mice were injected intravenously through the tail vein with cMLV (5****mg/kg Dox), cMLV(5****mg/kg PTX), 5****mg/kg Dox + 5****mg/kg PTX, or cMLV(5****mg/kg Dox + 5****mg/kg PTX). Three days after injection, tumors were excised, fixed, frozen, cryo-sectioned, and mounted onto glass slides. Frozen sections were fixed and rinsed with cold PBS. After blocking and permeabilization, the slides were washed by PBS and then incubated with TUNEL reaction mixture (Roche, Indianapolis, Indiana) for 1****h. For P-gp expression, the slides were stained after permeabilization with mouse monoclonal anti-P-gp antibody (Abcam, Cambridge, MA) for 1****h, followed by staining with Alexa488-conjugated goat anti-mouse immunoglobulin G (IgG) antibody (Invitrogen, Carlsbad, CA) and counterstaining with DAPI (Invitrogen, Carlsbad, CA). Fluorescence images were acquired by a Yokogawa spinning-disk confocal scanner system (Solamere Technology Group, Salt Lake City, UT), using a Nikon Eclipse Ti-E microscope. Illumination powers at 405, 491, 561, and 640 nm solid-state laser lines were provided by an AOTF (acousto-optical tunable filter)-controlled laser-merge system with 50****mW for each laser. All images were analyzed using Nikon NIS-Elements software. To quantify TUNEL and P-gp- positive cells, 4 regions of interest (ROI) were randomly chosen per image at ×2 magnification. Within one region, area of TUNEL, or P-gp-positive nuclei, and area of nuclear staining were counted by Nikon NIS-Element software. The data are expressed as % total nuclear area stained by TUNEL or P-gp in the region.

### Hematoxylin and Eosin staining of heart sections

Mice bearing 4T1 tumors were i.v. injected with 5****mg/kg Dox + 5****mg/kg PTX or cMLV(5****mg/kg Dox+5****mg/kg PTX). Three days after injection, heart tissues were harvested and fixed in 4% formaldehyde. The tissues were frozen, cut into sections, and mounted onto glass slides. The frozen sections were stained with hematoxylin and eosin. Histopathologic specimens were examined by light microscopy.

### Statistics

Differences between two groups were determined with Student’s *t* test. The differences among three or more groups were determined with a one-way ANOVA.

## Results

### In vitro efficacy study by XTT assay

**Figure 1:**
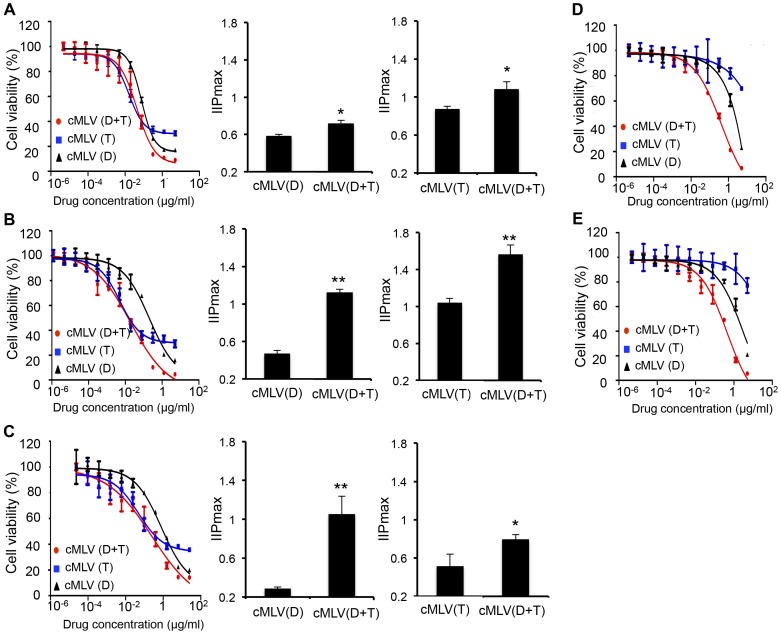
Overcoming drug resistance by codelivery of Dox and PTX via cMLVs (D: Dox; T: PTX). (A, B) *In vitro* cytotoxicity of cMLV(single drug) and cMLV(drug combinations) in B16 melanoma tumor cells (A) and 4T1 breast tumor cells (B). (C, D, E) *In vitro* cytotoxicity of cMLV(single drug) and cMLV(drug combinations) in drug-resistant JC cells (C), B16-R cells (D) and 4T1-R cells (E). IIP_Cmax_ was determined by incorporating three parameters (IC_50_, *D* and m) in the median effect model into the following equation: IIP_Cmax_  = log (1+(Cmax/IC_50_)^m^). Data are represented as mean ± SD (n = 3). Asterisks indicate statistical significance between two groups (*P < 0.05, **P < 0.01).

To achieve combination delivery of doxorubicin (Dox) and paclitaxel (PTX), a previously developed crosslinked multilamellar liposomal vesicle (cMLV) was used to incorporate PTX in the lipid membrane and encapsulate Dox in the aqueous core at a 1∶1 ratio to form cMLV(Dox+PTX) [30]. We chose this combination ratio because our previous study showed that it could induce synergy combination effect both *in vitro* and *in vivo* [31]. It has been reported that drug combinations can overcome drug resistance that would otherwise limit the potential application of various monotherapeutics [10]. To determine whether codelivery of Dox and PTX could overcome drug resistance, an *in vitro* cytotoxicity assay was performed at a wide range of concentrations of single drug-loaded or dual drug-loaded cMLVs. As shown in Figure 1A and 1B (left panel), both B16 cells and 4T1 cells developed drug resistance to single drug-loaded cMLVs, but this resistance was inhibited by applying the combined formulation, cMLV(Dox+PTX). The maximal cytotoxicity of single drug-loaded cMLV observed in these two tumor cells was between 60%–80%, while cells treated with dual drug-loaded cMLV(Dox+PTX) showed significantly more growth inhibition (∼95%).

To further confirm the efficiency of dual drug-loaded cMLVs in overcoming drug resistance, drug-resistant cell lines B16-R and 4T1-R were generated by continuously treating parental B16 or 4T1 with a high concentration of paclitaxel (5 µg/ml). Various concentrations of single drug-loaded cMLV and dual drug-loaded cMLV(Dox+PTX) were incubated with these two drug-resistant cell lines for 48****h, and the cytotoxicity was measured by a standard XTT assay. As shown in Figure 1D and 1E, both B16-R and 4T1-R cells showed a high tolerance when treated with cMLV(PTX) or cMLV(Dox), indicating that multidrug resistance had been developed in these cells. In contrast, cMLV(Dox+PTX) triggered significantly more cell death (90–100%) compared to that of single drug-loaded cMLVs, confirming that a codelivery system could overcome drug resistance induced by a high concentration of single drug. Furthermore, *in vitro* cytotoxicity studies demonstrated therapeutic efficacy of cMLV(Dox+PTX) in JC cells, a model drug-resistant tumor cell line, corroborating the weaker potency of single drug-loaded cMLVs compared to the dual drug-loaded cMLVs. As shown in Figure 1C (left panel), the maximal cytotoxicity of cMLV(Dox) and cMLV(PTX) was in the range of 60–70%, while peak cMLV(Dox+PTX) cytotoxicity was about 90% in JC cells.

IC_50_, which indicates drug concentration that causes 50% inhibitory effect on cell proliferation, can provide information on the efficacy of drugs. The IC_50_ values of the individual drugs and combined drugs through cMLVs in B16, 4T1 and JC cells are provided in Figure S1. However, it has also been reported that slope m, a parameter mathematically analogous to the Hill coefficient, may also have a significant effect on cytotoxicity [33], [34]. Therefore, a new model has been developed to evaluate drug activity by incorporating three parameters (IC_50_, drug concentration, and m) from the median effect model into a single-value IIP (potential inhibition) with an intuitive meaning, i.e., the log reduction in inhibitory effect [34]. Accordingly, to increase the trustworthiness of our experiment, IIP was used to evaluate the efficiency of dual drug-loaded cMLVs on cell viability. As shown in Figure 1A to 1C (middle and right panels), Dox and PTX in the dual drug-loaded cMLVs displayed a significantly larger IIP_Cmax_ value in the cell lines studied compared to that of the single drug-loaded cMLVs, indicating that combinatorial cMLVs were more potent in cancer treatment than single drug-loaded cMLVs.

### Cellular uptake study of doxorubicin and paclitaxel

**Figure 2:**
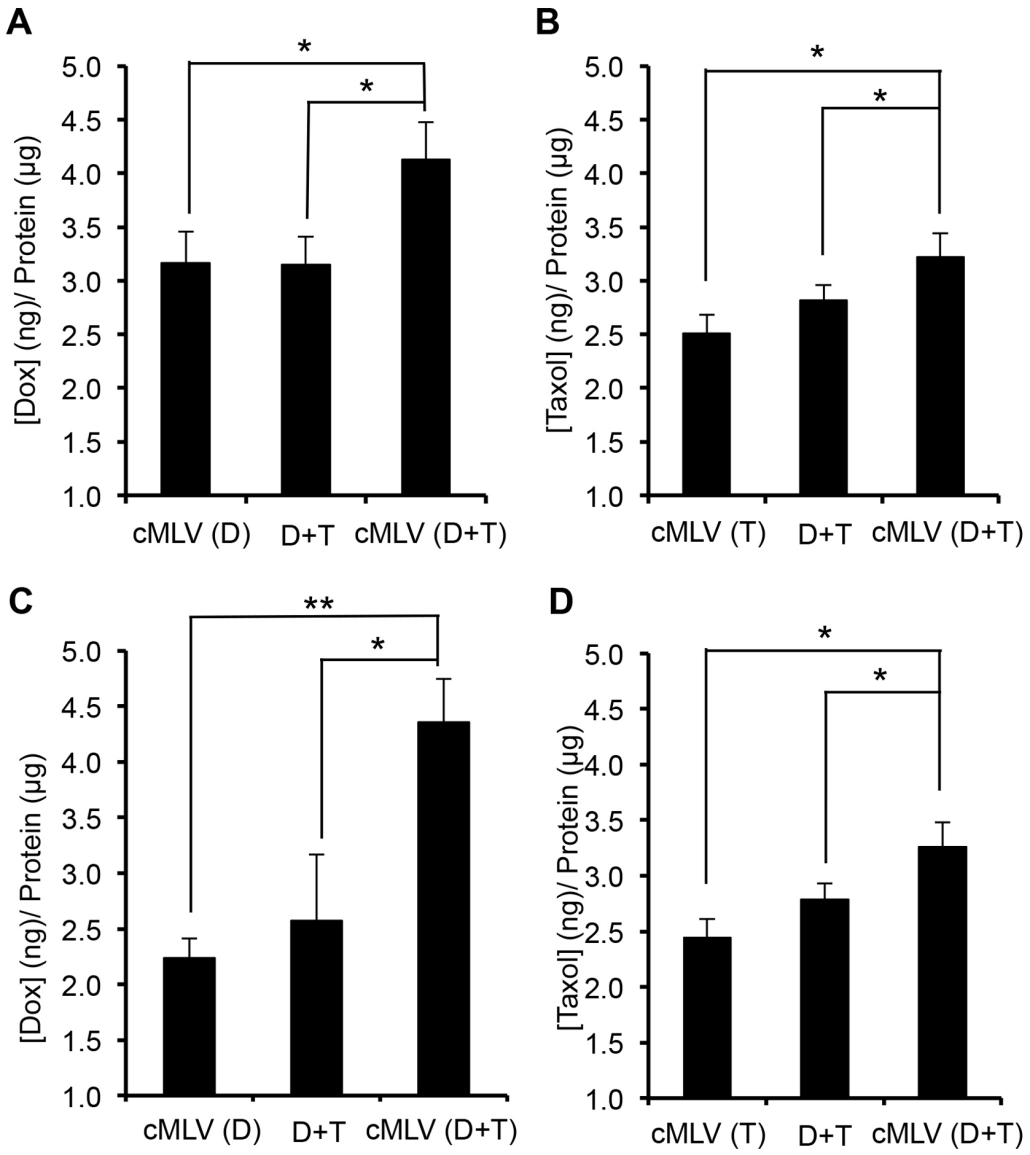
Cellular uptake of Dox and PTX (D: Dox; T: PTX). (A, B) Total cellular uptake of Dox (A) and PTX (B) into 4T1 cells. 4T1 cells were exposed to cMLV(D), cMLV(T), cMLV(D+T), and D+P in solution. The final concentrations of Dox and PTX were 1 µg/ml for each group. (C, D) Total cellular uptake of Dox (C) and PTX (D) in JC cells. JC cells were exposed to cMLV(D), cMLV(T), cMLV(D+T), and D+T. The final concentrations of Dox and PTX were 5 µg/ml for each group. The uptake of Dox and PTX was normalized to protein content measured with the BCA assay. All data are shown as the means of triplicate experiments from three different nanoparticle preparations. Asterisks indicate statistical significance between two groups (*P < 0.05, **P < 0.01).

To investigate the mechanism of enhanced cytotoxicity observed with cMLV combination therapy, we evaluated the effect of dual drug-loaded cMLVs on rates of drug influx/efflux in cells. The intracellular accumulation of Dox and PTX was examined by HPLC in 4T1 cells following exposure to Dox (1 µg/ml) and PTX (1 µg/ml) in cMLVs, both individually and in combination, and in JC cells with higher dose of Dox and PTX (5 µg/ml). After 3****h incubation, the extracellular medium was discarded, and intracellular drug (Dox or PTX) accumulation was quantitatively determined by drug concentration in the cell lysates, normalized by total cellular protein content of the cells. As seen in Figure 2A and 2B, cMLV(Dox+PTX) significantly increased both Dox and PTX accumulation in 4T1 cells compared to that of single drug-loaded cMLVs (*p* < 0.05), suggesting that combination treatments may overcome drug resistance. In addition, compared to the administration of drug in solution, cMLV combination treatment resulted in higher cellular accumulation of Dox and PTX, an outcome most likely resulting from the internalization of cMLVs by cells through endocytosis [30] and, consequently, effectively bypassing the P-gp efflux pumps. The enhanced cellular accumulation of drugs in dual drug-loaded cMLVs was also observed in drug-resistant JC cells (Figure 2C and 2D) compared to single drug-loaded cMLVs and drug combination in solution. These data suggest that cMLV(Dox+PTX) significantly enhanced the intracellular accumulation of anticancer drugs through mechanisms involving both combination treatment and nanoparticle delivery.

### Effect of codelivered nanoparticles on P-gp expression

**Figure 3:**
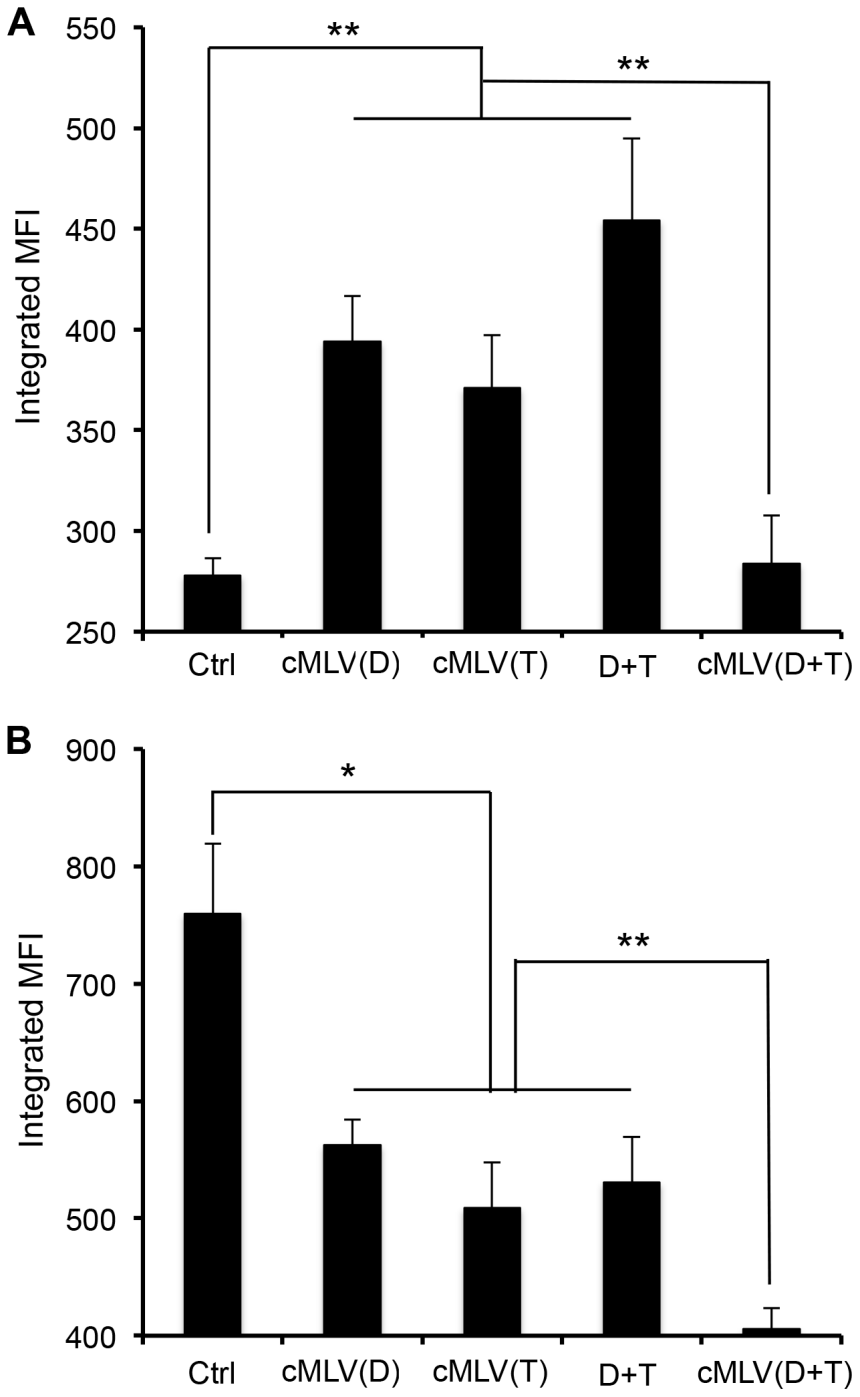
Effect of codelivered nanoparticles on P-gp expression (D: Dox; T: PTX). (A) 4T1 cells were exposed to empty cMLVs (Ctrl), cMLV(D), cMLV(T), cMLV(D+T), and D+T with the same concentration of Dox and PTX (1 µg/ml). (B) JC cells were exposed to empty cMLVs (Ctrl), cMLV(D), cMLV(T), cMLV(D+T), and D+T with the same concentration of Dox and PTX (5 µg/ml). P-gp expression was detected by P-gp-specific antibody *via* flow cytometry. Data are represented as mean ± SD (n = 3). Asterisks indicate statistical significance between two groups (*P < 0.05, **P < 0.01).

Having shown that dual drug-loaded cMLVs enhance cellular accumulation of drugs, we next sought to verify that this did, indeed, result from the modulation of membrane pumps, which are responsible for multidrug resistance. We first measured the expression of P-gp by flow cytometry in 4T1 cells treated with various nanoparticle formulations for 48 h to test if these cMLV formulations were responsible for altering P-gp involvement in multidrug resistance, along with decreased drug accumulation, in cells [3], [35]. As shown in Figure 3A, with the single drug-loaded cMLV treatment, the expression of P-gp (in terms of integrated mean fluorescence intensity) increased significantly in 4T1 cells (*p* < 0.01), possibly leading, in turn, to the development of drug resistance in 4T1 cells. However, dual drug-loaded cMLVs significantly inhibited expression of P-gp when compared to that of the single drug-loaded cMLVs and drug combination in solution (*p* < 0.01), suggesting that the combinatorial delivery of Dox and PTX *via* cMLVs could efficiently suppress P-gp expression, thereby overcoming MDR. We next investigated whether cMLV(Dox+PTX) could inhibit multidrug resistance in JC cells, which exhibit drug-resistant phenotype by overexpression of P-gp [32]. As shown in Figure 3B, the expression of P-gp decreased after 48 h of incubation with JC cells (*p* < 0.05) when treated with single drug-loaded cMLV, indicating that the nanoparticle drug delivery system could, at least partially, suppress MDR. However, the codelivery formulation of cMLV(Dox+PTX) significantly inhibited P-gp expression compared to that of single drug-loaded cMLVs and drug combination in solution (*p* < 0.01). Taken together, these results indicated that the codelivery of Dox and PTX via cMLVs could inhibit the expression of P-gp and increase cellular accumulation of drugs, leading to enhanced drug action in cells, including drug-resistant cells.

### Efficacy of dual drug-loaded cMLVs against a murine breast cancer model

**Figure 4:**
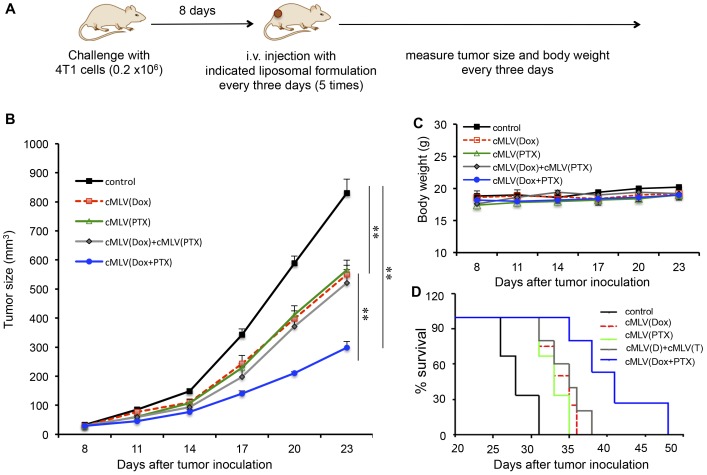
*In vivo* efficacy of drug combinations *via* cMLVs in a 4T1 tumor model. (A) Schematic diagram of the experimental protocol for *in vivo* 4T1 tumor study in BALB/c mice. (B) Tumor growth was measured after treatment with PBS (control, black solid line), cMLV (2 mg/kg Dox) (red dashed line), cMLV (2 mg/kg PTX) (green solid line), cMLV(2 mg/kg Dox)+cMLV(2 mg/kg PTX) (grey solid line), or cMLV (2 mg/kg Dox+2 mg/kg PTX) (blue solid line). Error bars represent standard error of the mean, n = 6 for each treatment group (**p < 0.01). (C) Average mouse weight loss over the duration of the experiment. (D) Survival curves for 4T1-bearing mice treated with PBS (black solid line), cMLV 2 mg/kg Dox) (red dashed line), cMLV (2 mg/kg PTX) (green solid line), cMLV(2 mg/kg Dox)+cMLV(2 mg/kg PTX) (grey solid line), or cMLV (2 mg/kg Dox+2 mg/kg PTX) (blue solid line). Survival end point was set when the tumor volume reached 1000 mm^3^. The survival rates were presented as Kaplan-Meier curves. The survival curves of individual groups were compared by a log-rank test.

It has been demonstrated that codelivery of Dox and PTX via cMLVs is able to overcome drug resistance *in vitro*. However, since the *in vivo* environment is considerably more complicated, it remains unknown if this effect could be translated to an animal cancer model. Therefore, in this experiment, a mouse breast tumor model was used to evaluate the therapeutic efficacy of dual drug-loaded cMLVs compared with that of single-drug liposomal formulations. At day 0, BALB/c mice were inoculated subcutaneously with 4T1 breast tumor cells. On day 8, mice bearing tumors were randomly sorted into six groups, and each group was treated with one of the following: PBS (control), cMLV(2 mg/kg Dox), cMLV(2 mg/kg PTX), cMLV(2****mg/kg Dox)+cMLV(2 mg/kg PTX), or cMLV(2 mg/kg Dox + 2 mg/kg PTX) every three days. Tumor growth and body weights were monitored until the end of the experiment (Figure 4A).

As shown in Figure 4B, mice in groups receiving cMLV(Dox), cMLV (PTX) or cMLV(Dox)+cMLV(PTX) exhibited tumor inhibition compared to those in the control group (*p* < 0.01). Even more significantly, cMLV(Dox+PTX) treatment induced a greater inhibition than that of cMLV encapsulating a single drug and that of cMLV(Dox)+cMLV(PTX), indicating that codelivery of Dox and PTX through single nanoparticle is essential for overcoming drug resistance (*p* < 0.01). As one indication of systemic toxicity, no weight loss was seen for the cMLV formulation over the duration of the experiment (Figure 4C). The *in vivo* efficacy of dual drug-loaded cMLVs against the 4T1 tumor model was further confirmed by a survival test. As shown in Figure 4D, the groups treated with cMLV(Dox), cMLV(PTX), or cMLV(Dox)+cMLV(PTX) had a prolonged lifespan compared to the control group, while the mice in the group treated with cMLV(Dox+PTX) had a significantly increased lifespan compared to the groups treated with single drug-loaded cMLVs and the group treated with cMLV(Dox) + cMLV(PTX) (*p* < 0.01).

### Histology study

**Figure 5:**
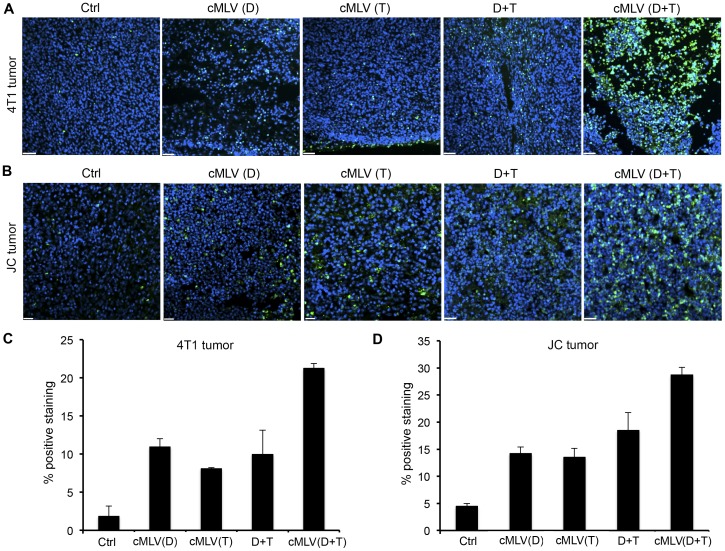
Effect of codelivered cMLVs on tumor apoptosis (D: Dox; T: PTX). (A, B) Mice bearing either 4T1 tumor (A) or multidrug-resistant JC tumor (B) were injected intravenously through the tail vein with cMLV (5 mg/kg Dox), cMLV (5 mg/kg PTX), 5 mg/kg Dox + 5 mg/kg PTX, or cMLV (5 mg/kg Dox+5 mg/kg PTX). Three days after injection, tumors were excised. Apoptotic cells were detected by a TUNEL assay (green), followed by nuclear costaining with DAPI (blue). Scale bar represents 50 µm. (C, D) Quantification of apoptotic cells in 4T1 (C) and JC (D) tumors. To quantify TUNEL-positive cells, 4 regions of interest (ROI) were randomly chosen per image at ×2 magnification. Within one region, area of TUNEL-positive nuclei and area of nuclear staining were counted. The data are expressed as % total nuclear area stained by TUNEL in the region. Data are represented as mean ± SD (n = 3).

To study the antitumor mechanism *in vivo*, a TUNEL assay was carried out to detect tumor cell apoptosis in tumors treated with Dox (5 mg/kg) and/or PTX (5 mg/kg) in various formulations for 3 days. As shown in Figure 5A and Figure 5C, 4T1 tumors treated with cMLV(Dox), cMLV(PTX), and Dox+PTX in solution showed significantly more apoptotic cells compared with controls (*p* < 0.01). The apoptosis index was also significantly higher in the cMLV(Dox+PTX)-treated group as compared with other groups (*p* < 0.05). Thus, the efficacy of cMLV(Dox+PTX) as an antitumor treatment could be explained by data suggesting increased tumor cell apoptosis. To further confirm the induction of cell apoptosis in treated groups, the TUNEL assay was performed in drug-resistant JC tumors treated with various formulations for 3 days. As shown in Figure 5B and 5D, cMLV(Dox), cMLV(PTX), and Dox+PTX induced more apoptotic cells compared to control JC tumors (*p* < 0.01). Dual drug-loaded cMLV-treated JC tumors showed a remarkably higher apoptosis index compared with other groups (*p* < 0.01), again confirming the enhanced antitumor activity of cMLV(Dox+PTX).

**Figure 6:**
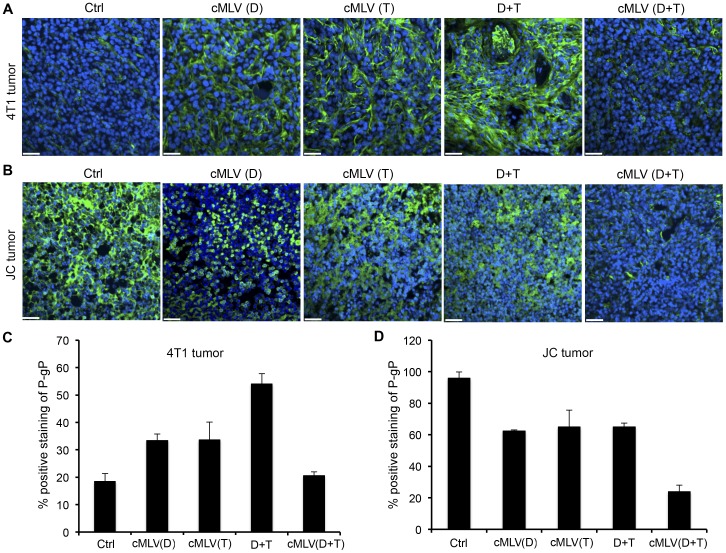
Effect of codelivered cMLVs on P-gp expression in tumors. (A, B) Mice bearing 4T1 tumor (A) and multidrug-resistant tumor JC (B) were injected intravenously through the tail vein with cMLV (5 mg/kg Dox), cMLV (5 mg/kg PTX), 5 mg/kg Dox + 5 mg/kg PTX, or cMLV (5 mg/kg Dox+5 mg/kg PTX). Three days after injection, tumors were excised, and stained by P-gp-specific antibody (green), followed by nuclear costaining with DAPI (blue). Scale bar represents 50 µm. (C, D) Quantification of P-gp-positive cells in 4T1 (C) and JC (D) tumors. To quantify P-gp-positive cells, 4 regions of interest (ROI) were randomly chosen per image at ×2 magnification. Within one region, area of P-gp-positive nuclei and area of nuclear staining were counted. The data are expressed as % total nuclear area that is P-gp-positive in the region. Data are represented as mean ± SD (n = 3).

To further investigate the innate characteristics of treated tumors, both 4T1 and JC tumor sections from each treatment group were analyzed for the expression of P-gp protein. As shown in Figure 6A, P-gp expression level was moderate in the control group. There appeared to be a significant enhancement of P-gp expression in the cMLV(Dox) and cMLV(PTX) groups, with an even more significant enhancement in Dox+PTX group compared to controls. However, a marked decrease was observed in the cMLV(Dox+PTX)-treated group when compared to the cMLV(Dox), cMLV(PTX), and Dox+PTX groups, as further confirmed by the quantification data in Figure 6C (*p* < 0.01). Interestingly, P-gp was very high in the JC tumor control group, as shown in Figure 6B. However, a significant decrease appeared in the cMLV(Dox), cMLV(PTX), and Dox+PTX groups, as further confirmed by the quantification data in Figure 6D (*p* < 0.05). An even more significant decrease of P-gp expression was seen in the cMLV(Dox+PTX) group (*p* < 0.01), indicating that dual drug-loaded cMLVs might be able to alter the innate characteristics of the multidrug-resistant tumor cells such as JC cells. Taken together, these data show that drug-loaded nanoparticles can partially bypass the P-gp efflux pumps to increase cellular uptake of Dox and PTX, sufficiently inducing cytotoxicity in cancer cells.

It has been reported that Dox treatment results in severe irreversible cardiotoxicity, leading to myocyte apoptosis [36]. In addition, cardiac toxicity, an unexpected clinical outcome of combinatorial Dox and PTX treatment, has been reported [37]. Therefore, systemic toxicity of free Dox+PTX and cMLV(Dox+PTX) was evaluated to determine whether codelived cMLVs could decrease this side effect of combination drug treatment. To accomplish this, a single intravenous dose of either Dox+PTX in solution or cMLV(Dox+PTX) was administered to mice bearing 4T1 tumors. Next, hematoxylin and eosin-stained cardiac tissue sections from each treatment group were examined (Figure S2). Treatment with free Dox (5 mg/kg) and PTX (5 mg/kg) in solution did cause cardiac toxicity, as indicated by myofibril loss, disarray, and cytoplasmic vacuolization. However, when cMLV(5 mg/kg Dox+5 mg/kg PTX) was administered under the same experimental conditions *via* cMLVs, no visible loss of myocardial tissue was observed.

## Discussion

Chemotherapeutics are crucial to combating a variety of cancers; however, clinical outcomes are always poor, as cancer cells develop a multidrug resistance (MDR) phenotype after several rounds of exposure to the chemotherapeutics. Many efforts have been made to develop a therapeutic strategy to overcome tumor MDR through the use of combined therapeutics to enhance the efficiency of systemic drug delivered to the tumor site and lower the apoptotic threshold. In this study, we have examined augmentation of therapeutic efficacy upon co-administration of Dox and PTX using a crosslinked multilamellar liposomal vesicle (cMLV) in breast cancer cells and drug-resistant JC cells. We demonstrated that combination therapy of Dox and PTX, especially when codelivered in cMLV formulations, was effective in enhancing the cytotoxicity in both wild-type and drug-resistant cells by elevating the cellular accumulation and retention of the drugs. We also showed that the dual therapeutic strategy efficiently suppressed tumor growth by enhancing apoptotic response.

P-glycoprotein (P-gp), a membrane-bound active drug efflux pump, is considered one of the most important mechanisms involved in MDR [3], [35]. As a result, growing interest has been shown in the development of nanoparticle drug delivery systems to overcome MDR. With their unique properties, nanoparticles are able to passively target the tumor mass through the enhanced permeability and retention (EPR) effect, enhancing the accumulation of chemotherapeutics at target sites [23], [24]. In addition, nanoparticles can enter cells through the endocytosis pathway, which is thought to be independent of the P-gp pathway, thus increasing the cellular uptake and retention of therapeutics in resistant cancer cells [26], [27]. Previously, we demonstrated the advantage of cMLVs in cancer therapy over conventional liposomal formulations based on their sustained drug release, enhanced vesicle stability and improved drug release, resulting in improved therapeutic activity with reduced systemic toxicity [30]. Further investigation of this novel liposomal formulation showed that it enable to translate the synergistic combination effect from in vitro to in vivo antitumor efficiency [31]. Moreover, cMLVs are internalized by tumor cells through caveolin-mediated endocytosis [30], suggesting that cMLVs could be an efficient drug carrier to overcome MDR. In this study, our *in vitro* and *in vivo* results demonstrated that the co-administration of Dox and PTX at the synergistic ratio (1∶1) *via* cMLVs efficiently suppressed P-gp expression in both wild-type and drug-resistant cancer cells.

In addition to nanodelivery, another potential strategy to overcome MDR has resulted from combining multiple drugs. For example, the combination of Dox and PTX in a cocktail is a standard anthracycline-taxane treatment regimen and was found to be efficacious in treating a variety of tumors by reducing the individual drug concentration that would otherwise be required to achieve cytotoxicity, thus overcoming drug resistance [13], [14], [15]. However, its clinical outcome was limited by the un-coordinated biodistribution of combined drugs [16], [17] and increase in cardiac cytotoxicity [37]. In this study, the pharmacokinetics of Dox and PTX was unified through the encapsulation of both drugs into a single cMLV particle, resulting in dual drug-loaded cMLVs which successfully reduced P-gp expression, increased the cellular accumulation of drugs, and enhanced cytotoxicity in cancer cells, including drug-resistant cells, as compared to single drug-loaded cMLVs. Moreover, combination therapy of Dox and PTX administered in cMLV formulations showed increased efficacy over cMLV monotherapy in the suppression of tumor growth by promoting apoptotic response *in vivo*.

## Conclusion

In summary, we have developed a multimodal therapeutic strategy to overcome tumor MDR by codelivery of Dox and PTX *via* a crosslinked multilamellar liposomal vesicle. We demonstrated that such combinatorial delivery system increased therapeutic efficacy by enhancing delivery efficiency to tumors and lowering the apoptotic threshold of individual drugs, thus overcoming drug resistance. The properties of cMLVs, such as improved stability and sustained release of drugs, enable the nanoparticles to sufficiently accumulate at tumor sites, subsequently entering tumor cells *via* endocytosis to release therapeutics, thus potentially bypassing the P-gp pathway to enhance cellular retention of therapeutics. Moreover, cMLVs enable multidrug delivery to the same action site, thereby lowering the tumor apoptotic threshold of individual therapeutics and potentially inhibiting the MDR. Taken together, this dual drug-loaded cMLV approach shows promise for reducing MDR in cancer therapeutics.

## Supporting Information

Figure S1 **IC50 values of cMLV(Dox), cMLV(PTX) and cMLV(Dox+PTX) in B16 melanoma, 4T1 breast tumor cells, or drug-resistant JC cancer cells.**
(TIF)

Figure S2 **Histologic appearance (hematoxylin and eosin staining) of heart tissues by light microscopy isolated on day 3 after a single intravenous injection of PBS (left), 5 mg/kg Dox+5 mg/kg PTX in solution (middle) and cMLV(5 mg/kg Dox+5 mg/kg PTX) (right).**
(TIF)
