# Supplementary figures and images for: Codelivery of Chemotherapeutics via Crosslinked Multilamellar Liposomal Vesicles to Overcome Multidrug Resistance in Tumor
Source: PLoS One. 2014 Oct 17;9(10):e110611. doi: 10.1371/journal.pone.0110611 (PMC4201570; doi:10.1371/journal.pone.0110611)

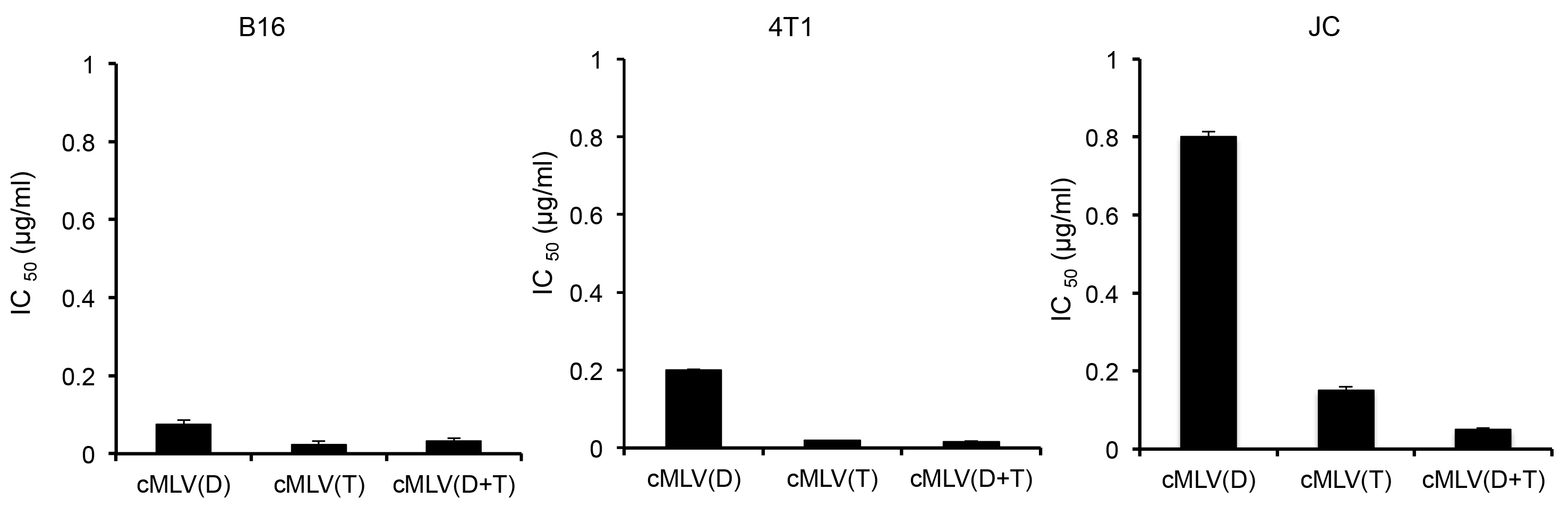

Supplement: Figure S1 — IC50 values of cMLV(Dox), cMLV(PTX) and cMLV(Dox+PTX) in B16 melanoma, 4T1 breast tumor cells, or drug-resistant JC cancer cells. (TIF) [file pone.0110611.s001.tif]

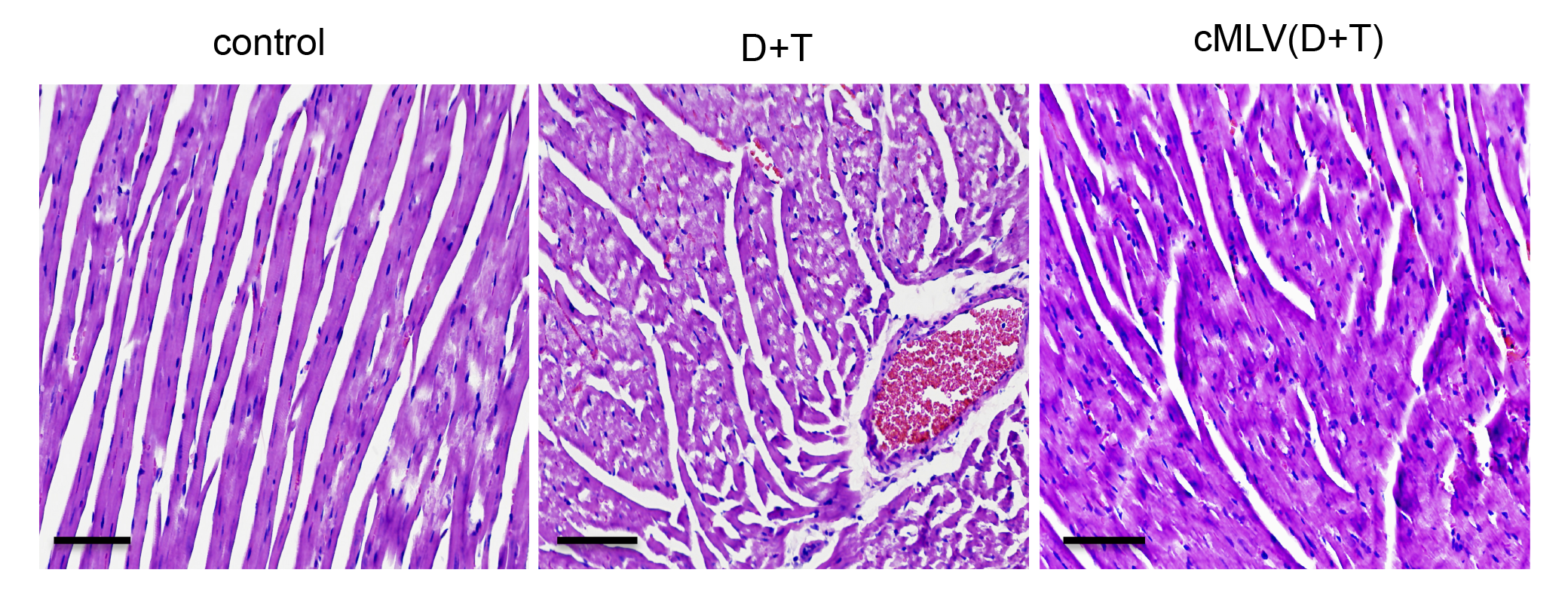

Supplement: Figure S2 — Histologic appearance (hematoxylin and eosin staining) of heart tissues by light microscopy isolated on day 3 after a single intravenous injection of PBS (left), 5 mg/kg Dox+5 mg/kg PTX in solution (middle) and cMLV(5 mg/kg Dox+5 mg/kg PTX) (right). (TIF) [file pone.0110611.s002.tif]
